# A High‐Energy Density Li‐Ion Hybrid Capacitor Fabricated from Bio‐Waste Derived Carbon Nanosheets Cathode and Graphite Anode

**DOI:** 10.1002/gch2.202200082

**Published:** 2022-08-09

**Authors:** Katchala Nanaji, Samhita Pappu, Srinivasan Anandan, Tata N. Rao

**Affiliations:** ^1^ Centre for Nanomaterials International Advanced Research Centre for Powder Metallurgy and New Materials (ARCI) Hyderabad 500005 India

**Keywords:** activated carbon sheets, cathode materials, graphite, high energy density, Li‐ion capacitors

## Abstract

The Li‐ion hybrid capacitor (LIHC) system explores the possibility of achieving both high energy and power density in a single energy storage system with an intercalation anode and capacitive cathode. However, to achieve a high power and energy‐based system, the properties of the cathode electrode material are vital. Here, bio‐waste plant stem‐derived activated porous carbon is explored as a cathode for LIHC application. A specific surface area of 1826 m^2^ g^−1^, enhanced degree of crystallinity, and graphitization results for porous carbon from activation by potassium hydroxide. When employed as supercapacitor material, the device exhibits good rate capability, energy, and power attributes with a specific capacitance of 116 F g^−1^ (1 A g^−1^). Simultaneously when tested for LIHC application the formulated device shows good capacity retention for 2500 cycles with a high energy density of 125 Wh kg^−1^ at a power density of 69 W kg^−1^. The work demonstrates unique, cost‐effective strategy to develop a crystalline high surface area carbon from any such bio‐waste sources to be employed as potential electrodes for energy storage applications.

## Graphical abstract

Activated carbon with a specific surface area of 1826 m^2^ g^−1^, and graphene sheet‐like morphology is prepared by KOH activation. The carbon nanosheets are demonstrated for a Li‐ion capacitor as a high‐rate cathode material by employing a commercial graphite anode. The Li‐ion capacitor device exhibits a maximum high energy and power densities of 125 Wh kg^−1^ and 2083 W kg^−1^ respectively.

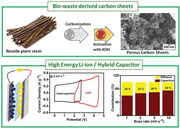

## 1 Introduction

The rapidly growing electric vehicle technologies and next‐generation consumer power systems necessitate the development of high energy and power‐based storage systems with extended lifespans.^[^ ^1^, ^2^ ^]^ Batteries are one such system which can reach ≈200 Wh kg^−1^ energy density via lithiation and de‐lithiation (in case of Li‐ion batteries) of electrolytes ions in the electrode material. Supercapacitors, on the other hand, are characterized by a high power density, long cycle life (>10^5^ cycles), good rate, and easy maintenance, making them suitable for high‐power applications.^[^ ^1^ ^]^ Nevertheless, the low energy density (<10 Wh kg^−1^) restricts their individual commercialization.^[^ ^3^ ^]^ Hence, the quest for a single energy storage system equipped with both high energy and power characteristics along with good rate and stability seems insatiable. Early 2000s has seen the advent of Li‐ion hybrid capacitors (LIHC) which conjugates a high‐capacity anode capable of high charge acceptance with reversible Li‐ion insertion and de‐insertion and a high surface area porous cathode with efficient ion adsorption properties exhibiting electrical double layer charge (EDLC) storage behavior.^[^ ^4^, ^5^, ^6^ ^]^ This coalesces of a battery‐type anode with high energy density, and supercapacitor cathode with high power density is expected to integrate the complementary characteristics of these two devices. The charge storage mechanism in LIHCs is different from that of conventional batteries and supercapacitors. Upon charging the system above its open‐circuit voltage (OCV), the $PF_{6}^{-}$ and Li^+^ ions from the electrolyte get adsorbed on the cathode and intercalate on the anode, respectively. However, when the system is discharged below its OCV, the de‐intercalated $PF_{6}^{-}$ ions gets desorbed on the cathode and the reverse process takes place during charging.^[^ ^7^, ^8^, ^9^, ^10^ ^]^ Various metal alloys, oxides, and carbon materials are explored as electrodes for LIHCs. Specific to the carbon‐based anode counterparts, graphene, graphite, hard carbon, soft carbon, carbon nanofibers, etc., are studied.^[^ ^11^, ^12^, ^13^ ^]^ The LIHCs with graphene//activated carbon (AC), graphite//AC, hard carbon//AC have shown 80–150 Wh kg^−1^ energy densities at 150–65 W kg^−1^ power densities.^[^ ^14^, ^15^, ^16^ ^]^ One of the reports investigates the electrochemical performance of natural graphite, artificial graphite, and hard carbon as anodes for LIHCs.^[^ ^16^ ^]^ Solid electrolyte interface (SEI) formation is essentially required by the carbon anodes to protect the consumed electrolyte decomposition and continue Li‐ion insertion and de‐insertion.^[^ ^17^, ^18^ ^]^ In general, hard carbons are found to have superior initial irreversible capacity loss resulting from the energy consumed during the SEI formation.^[^ ^19^ ^]^ Nevertheless, the hard carbons are also proven to have better rate capability and stability than other carbon anodes. In another report, the electrochemical performances of petroleum coke‐derived soft carbon and graphite anodes were examined. The petroleum coke‐derived soft carbon showed superior cycle stability similar to EDLCs while the graphite exhibited high charge storage properties.^[^ ^20^, ^21^ ^]^ The unaltered fact of high energy battery anode materials leads to the rationale understanding that the energy and power densities of LIHCs mainly depend on the cathode counterparts due to the asymmetric device fabrication and huge difference in the ratio of specific capacity of cathode:anode.^[^ ^1^ ^]^ In this context, different types of carbon materials have been extensively studied as cathode materials for LIHCs due to their high conductivity, ease of availability, tunable physical and chemical properties, and high economic value.^[^ ^1^ ^]^ Among these, bio‐waste‐derived carbon materials are lately attracting wide interest owing to their cost‐effectiveness, ease of availability, and economic importance. Many bio‐waste‐derived carbons have been researched quite extensively for supercapacitor applications both in symmetric and asymmetric configurations. But the use of the same in all carbon‐based LIHCs is limited.

In the present work, we report an activated carbon derived from the *Roselle* plant stem as cathode for LIHCs. *Roselle* is a flowering plant belonging to the family of *Hibiscus*. The flowers and leaves of the plant are found to be edible, while the stem is used for the production of blast fiber which is further employed for the production of woven fabrics, rope, yarn, carpets, etc. In line with the other potential applications, in the current work, bio‐waste‐derived plant stems have been carbonized and activated in potassium hydroxide (KOH) to obtain graphene‐like porous carbon nanosheets networks with high surface area and conductivity. The work shows the potential utility of these bio‐waste‐derived carbon nanosheets in both supercapacitors and LIHC applications. A specific capacitance of 116 F g^−1^ was observed with the designed activated carbon‐based supercapacitor at 1 A g^−1^. Further, when employed as efficient cathode for LIHCs, the formulated device has shown a high energy density of 125 Wh kg^−1^ at a power density of 69 W kg^−1^ along with good rate capability and stability.

## 2 Results and Discussions

**Figure 1:**
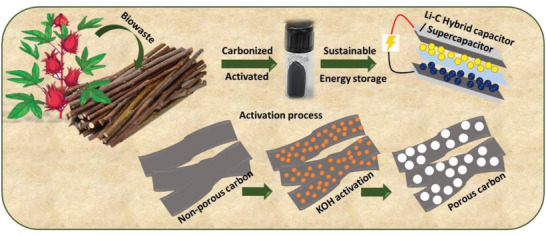
Bio‐waste plant stem derived activated porous carbon nanosheets synthesis by a schematic representation.

Activated porous carbon nanosheets were prepared from bio‐waste‐derived plant stems involving the process of carbonization, followed by activation in KOH at 900 °C for 1 h in Ar atmosphere. A detailed description of the synthesis is mentioned in the Experimental Section, “Bio‐Waste Plant Stem Derived Porous Carbon Nanosheet Synthesis”. **Figure** **1** shows the conversion of bio‐waste plant stem to activated porous carbon nanosheets by a schematic representation.

**Figure 2:**
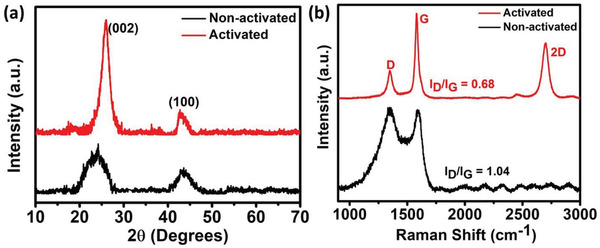
a) XRD, and b) Raman analyses of non‐activated and activated porous carbon nanosheets.

The crystal structure of both activated and non‐activated carbons was studied by the XRD technique shown in **Figure** **2**a. Both the materials show a characteristic peak at around 24–26°, corresponding to the (002) plane of graphitic carbon (Figure 2a).^[^ ^7^ ^]^ Attributed to the formation of ordered turbostratic carbon and increased degree of graphitization with improved conductivity, activated carbon shows a slight shift towards the right side with increased peak intensity.^[^ ^22^ ^]^ Additionally, a smaller peak at ≈43° is indexed to the (100) plane of carbon.^[^ ^7^ ^]^ Figure 2b shows the Raman spectra of both the activated and non‐activated carbons in the range of 900–3000 cm^−1^ with D and G‐bands positioned at 1350 and 1582 cm^−1^ respectively. The sp^2^ hybridized graphitic layers and tangential vibration of carbon atoms symbolic of their degree of graphitization and conductivity is indicated by the G‐band.^[^ ^3^ ^]^ In contrast, the D‐band is representative of the defects in the edge planes and graphitic layers. It can be observed from Figure 2b, that an improvement in the number of sp^2^ hybridized carbon atoms is exhibited by the reduction in D‐band and enhancement in G‐band intensity after activation. In comparison, almost the same intense D and G‐bands are observed in the non‐activated carbon material. The ratio of I_D_/I_G_ explains the nature of carbon materials. A lower I_D_/I_G_ represents a high degree of graphitization, while complementarily; higher I_D_/I_G_ indicates an increased degree of disorderliness in the material. As can be observed from Figure 2b, a lower I_D_/I_G_ (0.68) ratio of activated porous carbon nanosheets not only implies their higher conductivity and graphitization in consistent with the XRD results but also highlights the pivot role of KOH activation agent in creating a large number of pore edges and ripples easing adsorption and desorption of the electrolyte ions on the carbon nanosheets. Apart from this, a 2D band at 2650 cm^−1^ is observed for the activated carbon material, implying the presence of few‐layer graphene‐like sheets.

**Figure 3:**
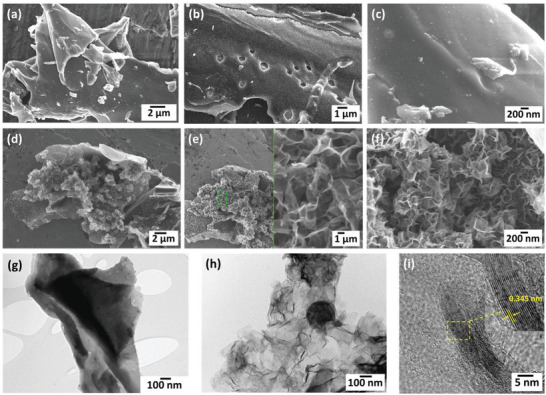
FESEM analysis of a–c) non‐activated carbon, d–f) activated porous carbon, and TEM, HRTEM analysis of g) non‐activated carbon, and h,f) activated porous carbon, respectively.

The morphological and microstructural analyses of the activated and non‐activated carbons were analyzed by FESEM, TEM, and HRTEM characterization tools, as shown in **Figure** **3** below. Figure 3a–c shows the FESEM morphology of the non‐activated carbon with a smooth plate‐like structure. The activation of the same with KOH as the pore‐inducing agent has resulted in surface roughening with a lot of porous nanosheet formation (Figure 3d–f). These porous nanosheets ease the intercalation and de‐intercalation of the electrolyte ions, reduce the diffusion path lengths, and are expected to provide more active sites for charge storage. Further, the microstructure of both non‐activated and activated carbons was studied by TEM and HRTEM analysis. Figure 3g shows the TEM image of non‐activated carbon having a smooth surface with no specific morphology in corroboration with the FESEM analysis. Subsequently, the activated carbon shows ultrathin, porous wrinkled sheet‐like morphology exhibiting a high surface area in comparison to the non‐activated one (Figure 3h). Also, the HRTEM analysis of the activated carbon shown in Figure 3i displays the presence of multiple graphene sheets with a d‐spacing of 0.345 nm.^[^ ^23^ ^]^

**Figure 4:**
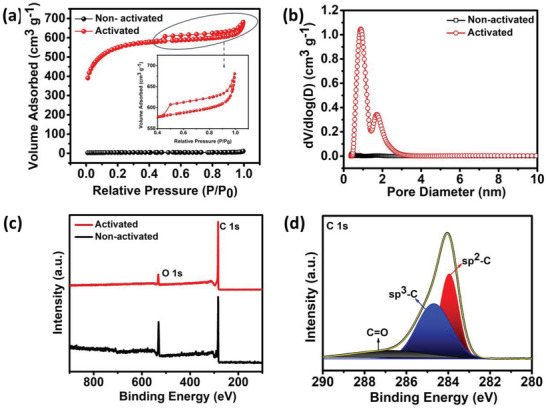
a) BET isotherm, b) pore size distribution analysis of non‐activated and activated carbon, c) XPS survey scan, and d) C 1s wide scan spectra of activated carbon.

The specific surface area (SSA) of the developed active materials was analyzed by carrying out the BET analysis as displayed in **Figure** **4**a,b. The activated carbon material exhibit an H2‐type hysteresis loop within the relative pressure region of 0.5–0.9 *P*/*P*_o_ with a type IV isotherm curve.^[^ ^24^ ^]^ While the non‐activated carbon was limited to a very low SSA of 15 m^2^ g^−1^, the activated porous carbon nanosheets have an average pore diameter of 2.2 nm, pore volume of 1.39 cm^3^ g^−1^, and a high SSA of 1826 m^2^ g^−1^. This high SSA after KOH activation is anticipated to drastically improve the charge storage performance in comparison to the non‐activated one. Further, the pore size distribution analysis has revealed a combination of both mesopore and micropore arrangement wherein the micropores participate in the charge storage and the mesopores are expected to be involved in the diffusion of charge carriers. The detailed analysis of the various functional groups attached to the surface and edge planes of the carbon material was carried out by employing the XPS technique. Figure 4c shows the survey scan spectra of both the non‐activated and activated carbons indicating the presence of C 1s and O 1s peaks. Further, Figure 4d displays the wide scan spectra of activated C 1s. The spectra are deconvoluted into three peaks showing the presence of keto functional groups (C=O) at 287 eV, sp3 hybridized carbon at 284.7 eV, and sp^2^ hybridized carbon at 283.9 eV, respectively.^[^ ^25^ ^]^

**Figure 5:**
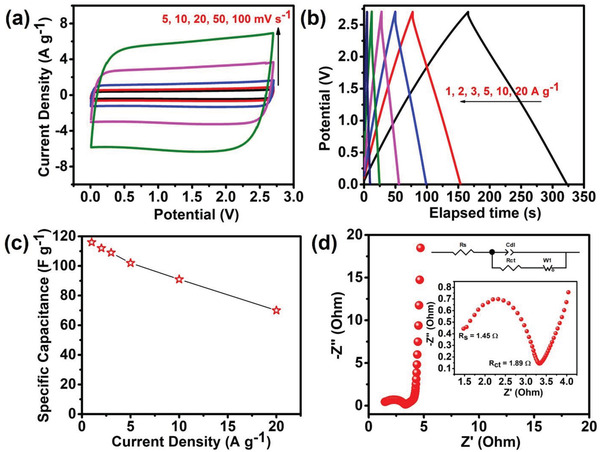
a) CV, b) CD, c) rate capability, and d) Nyquist plot analysis of the symmetric supercapacitor in organic electrolyte.

Due to the better structural, morphological, and surface porosity properties, the developed active materials were tested for their electrochemical characteristics for supercapacitor application, followed by LIHC application. Figure S1, Supporting Information, shows the individual CVs from 5 to 100 mV s^−1^ and CD from 1 to 50 A g^−1^ of both non‐activated and activated carbons, respectively. Both materials, within a voltage range of −1 to 0 V exhibit a rectangle CV indicative of EDLC charge storage. The peak shape is stable even at a high scan rate of 100 mV s^−1^, indicating that the material is capable of high rate applications. However, due to the high degree of graphitization, improved conductivity, crystallinity, and SSA, activated porous carbon nanosheets were found to have enhanced charge storage capabilities than the non‐activated material. As calculated from the CD analysis, the maximum observed specific capacitance (*C*_sp_) was 267 F g^−1^ (1 A g^−1^) for the activated carbon while the non‐activated carbon was limited to 103 F g^−1^ at same current density. The full potentiality of the activated carbon was further explored by the design of a symmetric supercapacitor in an organic electrolyte (1 M TEABF_4_ in acetonitrile (ACN)) owing to its excellent electrochemical characteristics in three‐electrode system. **Figure** **5**a shows the perfect rectangular CV curves within an electrochemical window of 0–2.7 V. The excellent electrode kinetics of the materials is displayed by the unaltered shape of the CV curve even at a high scan rate of 100 mV s^−1^. Further, the CD profiles exhibited in Figure 5b are perfectly triangular without any iR drop, showing EDLC charge storage phenomena. The *C*_sp_ calculated from the CD curve at 1 A g^−1^ was observed to be 116 F g^−1^. Also, the rate capability analysis revealed 60% capacitance retention with a *C*_sp_ of 70 F g^−1^ at 20 A g^−1^ (Figure 5c). Additionally, series (*R*_s_) and charge transfer (*R*_ct_) resistances were investigated by the Nyquist plot analysis respectively. An *R*_s_ of 1.45 Ω and a low *R*_ct_ of 1.89 Ω were observed, stating the excellent diffusion kinetics of the assembled device. Further, the capacitive nature of the active material was displayed by the straight line parallel to the y‐axis in the low‐frequency region (known as Warburg impedance).^[^ ^26^ ^]^

**Figure 6:**
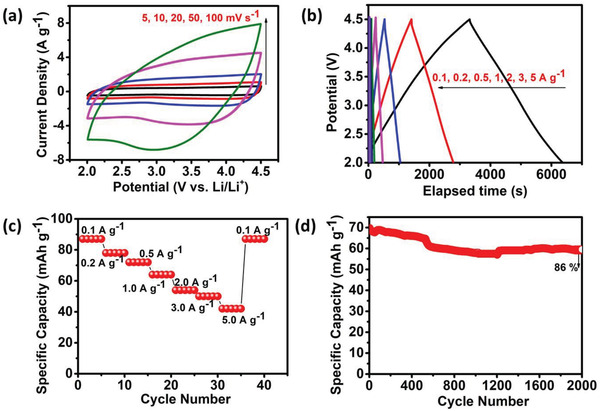
a) CV, b) CD, c) rate capability profile, and d) cycle stability at 0.5 A g^−1^ versus Li/Li^+^ ion of the activated carbon.

The work was further extended to explore the electrochemical characteristics of the material in LIHC application, owing to the excellent electrochemical performance of the developed active material in supercapacitors. One of the essential properties to be a suitable capacitive cathode for LIHC are high surface area and porosity. Hence, the high surface area activated carbon (1826 m^2^ g^−1^) with a pore volume of 1.39 cm^3^ g^−1^ was studied for its Li adsorption and desorption properties. The cathode half‐cell was analyzed versus Li/Li^+^ as the counter and reference electrode using the CV technique within 2.0–4.5 V potential window (**Figure** **6**a). CV shows near rectangular curves at scan rates from 5 to 100 mV s^−1^, demonstrating the capacitive nature of active material. The specific capacity was calculated from the CD analysis (Figure 6b), which translates the observation from CV with triangular‐shaped curves leading to a *C*_sp_ of 87 mAh g^−1^ at 0.1 A g^−1^ current density. Figure 6c showing the rate capability analysis indicates that the material is highly stable when tested at low to high and again low current densities attributing to the excellent reversible electrochemical behavior of the material. Further, the cycle stability analysis in Figure 6d shows almost 86% of specific capacity retention when cycled at 0.5 A g^−1^ for 2000 cycles, yet again proving its better reversibility and stability for LIHC application. Subsequently, the commercial graphite was tested for its anode potentiality within a potential range of 0.1–3.0 V versus Li/Li^+^ as displayed in Figure S2, Supporting Information. Figure S2a, Supporting Information, shows the CV profile indicating two cathodic peaks around 0.18 and 0.02 V versus Li/Li^+^ and anodic peaks at 0.20 and 0.26 V versus Li/Li^+^ corresponding to the LiC_x_ phase formations by Li‐ion intercalation and de‐intercalation at 0.1 mV s^−1^ scan rate respectively.^[^ ^27^ ^]^ Followed by, Figure S2b, Supporting Information, shows the CV scans from 0.1 to 2 mV s^−1^ scan rates. As observed from the CV scans, a broad peak at ≈0.87 V corresponds to the thin SEI layer formation due to the irreversible decomposition of electrolyte ions on the electrode surface.^[^ ^27^ ^]^ Further, the *C*_sp_ obtained from the CD analysis (Figure S2c, Supporting Information) shows an irreversible capacity loss of 19% in the 1st cycle with an initial capacity of 528 mAh g^−1^ at 0.1 A g^−1^. Followed by which a *C*_sp_ of 421 mAh g^−1^ was observed at the end of 5th cycle. At higher current densities of 0.2, 0.5, 1, 2 A g^−1^, the reversible specific capacities were found to be 398, 228, 115, and 46 mAh g^−1^, respectively. Consequently, the rate capability analysis of the commercial graphite anode exhibiting its good capacity retention when tested at both low and high current densities is exhibited in Figure S2d, Supporting Information.

Followed by the half‐cell electrochemical analysis, a full LIHC was assembled using activated carbon nanosheets and commercial graphite in 1 M LiPF_6_ EC:DEC electrolyte. An appropriate mass balance between cathode (activated carbon nanosheets) and anode (commercial graphite) was important to achieve charge balance between the cathode and anode, and to attain high efficiency in 1 M LiPF_6_ EC:DEC electrolyte. Therefore, Li‐Ion Capacitor was fabricated with activated carbon nanosheets to commercial graphite at the mass ratio of 4:1 and the ratio was taken based on the half‐cell performance of the cathode and anode versus Li/Li^+^.

(1) $$\begin{array}{c} \frac{\text{​}m_{+}}{m_{-}}=\frac{c_{-}}{c_{+}} \end{array}$$

**Figure 7:**
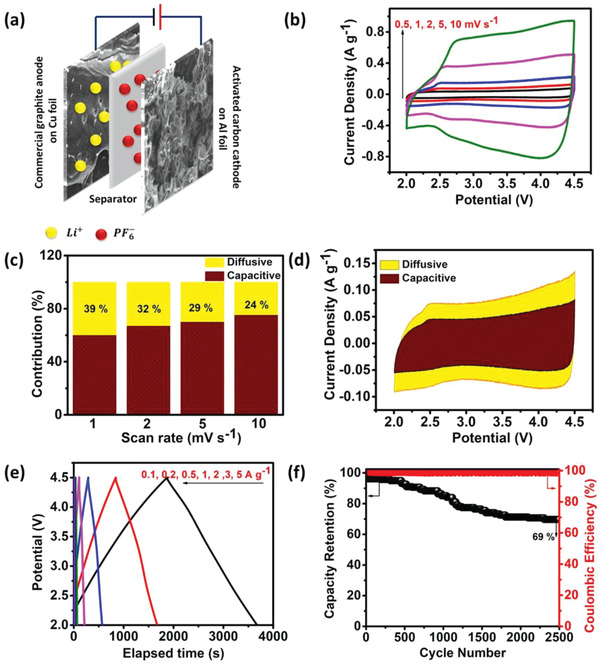
a) LIHC schematic, b) CV, c) capacitive and diffusion contributions at different scan rates, d) charge separation at 1 mV s^−1^, e) CD, and f) cycle stability at 1 A g^−1^

Where, *m*_+_(g) and *m*_+_ (g) are the masses of the positive and negative active electrode materials, respectively. Whereas, *c*_+_(mAh g^−1^) and *c*_−_(mAh g^−1^) are the gravimetric specific capacities of both positive and negative electrodes, respectively in the half‐cell configuration versus Li/Li^+^. The schematic representation of LIHC is shown in **Figure** **7**a. and the Figure 7b shows the CV within 2.0–4.5 V of LIHC from 0.5 to 10 mV s^−1^ exhibiting a non‐rectangular shape inferring a combination of capacitive contribution from the cathode and faradaic contribution from the anode electrodes, respectively. Further, “Trasatti” analysis was employed to individually analyze the combination of both capacitive and diffusive contributions at various scan rates.^[^ ^7^ ^]^ According to the Power law, it is understood that the current (*i*) is proportion to scan rate (*v*).^[^ ^7^ ^]^

(2) $$\begin{array}{c} i=av^{b} \end{array}$$

Where *a*,*b* are defined constants. “*b*” is obtained from the slope of the plot of log (i) versus log (v) ranging between 0.57–0.89, asserting both capacitive and diffusive contributions to the overall charge storage. Further, the quantification of the individual contributions was done by using the equation:

(3) $$\begin{array}{c} i\left(V\right)=k_{1}v+k_{2}v^{1/2} \end{array}$$

Where “*i*” is the current at a particular voltage, *k*_1_ *v* is the capacitive component and *k*_2_ *v* ^1/2^ is the diffusive component, respectively. From the slope and intercept of Figure S3b, Supporting Information, the quantified capacitive and diffusion contributions were obtained, and Figure 7c shows the bar graph representation of the corresponding contributions at different scan rates. It can be observed that with increasing scan rates, the capacitive contribution is dominant, attributing to the faster charge transfer kinetics of the highly porous activated carbon. Following, Figure 7d shows the 39% diffusive and 61% capacitive charge separation at 1 mV s^−1^ scan rate. Further, the specific capacity of the assembled LIHC was calculated from the CD analysis (Figure 7e), indicating an almost negligible IR drop with a *C*_sp_ of 50 mAh g^−1^ (72 F g^−1^) at 0.1 A g^−1^ by considering the mass of both electrodes respectively. The cyclic performance of the LIHC was carried at a current density of 1 A g^−1^ for 2500 cycles. At the end of 2500 cycles, specific capacity retention of ≈70% was obtained for the cell with 98% Coulombic efficiency. A slightly lower capacity retention property can be associated with the unwanted side reaction with the electrolyte counterpart and subsequent growth of thick SEI layer on the graphite electrode.^[^ ^28^ ^]^

**Figure 8:**
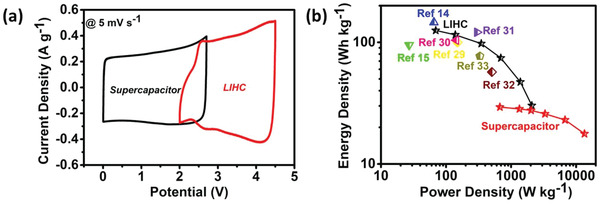
a) Comparative CVs of the assembled supercapacitor and LIHC, and b) Ragone plot with comparative literature reported data.

Simultaneously, Equations S3, S4, S6, and S7, Supporting Information were employed to estimate the gravimetric energy and power densities of both supercapacitor and LIHC (Supporting information). **Figure** **8**a shows the comparative CV at 5 mV s^−1^ of the formulated supercapacitor and LIHC with the activated porous carbon. Figure 8b displays the Ragone plot indicating the energy and power densities of the assembled devices in comparison to the literature reports. A power density of 673 W kg^−1^ at an energy density of 29 Wh kg^−1^ was achieved by the supercapacitor assembled with activated carbon nanosheets. And a noticeable retention of 17.7 Wh kg^−1^ energy density was observed even at a high power density of 13.4 k W kg^−1^. Similarly, a high energy density of 125 Wh kg^−1^ at a power density of 69 W kg^−1^ was attained owing to the excellent conjunction of battery anode and supercapacitor cathode. Further, the designed LIHC could still sustain an energy density of 30 Wh kg^−1^ with an increase in the power density to 2083 W kg^−1^. The obtained energy and power density of LIHC at different current densities have been compared and found to be on par with the literature‐reported values as presented in Figure 8b and **Table** **1**.^[^ ^14^, ^15^, ^29^, ^30^, ^31^, ^32^, ^33^ ^]^ The literature report with graphite//activated carbon LIHC reports a superior energy density of 146 Wh kg^−1^ at a power density of 65 W kg^−1^.^[^ ^14^ ^]^ This superior energy density is attributed to the wide electrochemical window of 1.5–5.0 V in comparison to the present work. While, some other reports such as Carbon from olive pit//activated olive pit,^[^ ^29^ ^]^ Carbon from sisal fiber//activated carbon from sisal fiber,^[^ ^30^ ^]^ LTO//Rice husk derived carbon,^[^ ^32^ ^]^ etc., report a high SSA greater than 1826 m^2^ g^−1^ nevertheless lack in terms of energy and power densities owing to the smaller electrochemical windows.

**Table 1:** Comparative literature report on electrochemical performance of carbon‐based LIHC in organic system

Device configuration (Anode//Cathode)	specific surface area [m^2^ g^−1^] of Cathode material	Voltage [V]	Energy density [Wh kg^−1^]	Power density [W kg^−1^]	Reference
Graphite//activated carbon	1402	1.5–5.0	146	65	[14]
Graphene//activated carbon	–	2.0–4.0	95	27	[15]
Carbon from olive pit//activated olive pit	2225	1.5–4.2	100	150	[29]
Carbon from sisal fiber//activated carbon from sisal fiber	3104	2.0–4.0	104	143	[30]
Hard carbon//biomass carbon	1795	1.7–4.2	121	300	[31]
LTO//rice husk‐derived carbon	2303	1.0–3.0	57	507	[32]
*N*‐doped nanoboxes//carbon nanosphere	1768	2.0–4.2	77	331	[33]
Commercial graphite//activated carbon activated carbon// activated carbon	1826	2.0–4.5 0–2.7	125 29	69 673	LIHC (This work) Supercapacitor (This work)

## 3 Conclusion

In the present work, bio‐waste‐derived activated carbon nanosheets were chosen as the cost‐effective and efficient source of carbon. The structural, morphological, and surface properties were tailored by KOH activation to improve the crystallinity, surface area, and electrochemical properties. With a high SSA of 1826 m^2^ g^−1^, lower *I*_D_/*I*_G_ value of 0.68, and graphene sheet‐like morphology, activated carbon was found to be superior in all characteristics. The material explored for its supercapacitor properties showed a high specific capacitance of 116 F g^−1^ at 1 A g^−1^. Also, a power density of 673 W kg^−1^ at an energy density of 29 Wh kg^−1^ was reported. In quest of a single energy storage system for high energy and power application, the developed activated carbon was studied along with a commercial graphite for LIHC application showcasing a device‐specific capacity of 50 mAh g^−1^ (72 F g^−1^) at 0.1 A g^−1^ current density. The device was found to be stable for as long as 2500 cycles with 69% capacity retention. Further, maximum high energy and power densities of 125 Wh kg^−1^ and 2083 W kg^−1^ were also attained. Such fine energy and power density attributes along with good stability of the LIHC are accredited to the fully graphitized, high surface area activated sheet‐like carbon. It is believed that the above work provides an appropriate, cost‐effective strategy to activate and convert any such bio‐waste‐derived carbon to highly crystalline, graphitic, porous nanosheet structures with high surface area aimed for better charge storage abilities.

## 4 Experimental Section

### Bio‐Waste Plant Stem Derived Porous Carbon Nanosheet Synthesis

Porous carbon nanosheets were developed by the carbonization and activation of bio‐waste plant stems derived from the *Rosella* plant from an Indian village (Kummarilova, Tuni). Initially, the obtained bio‐waste stems were cut into tiny pieces, washed with DI water followed by drying at 100 °C overnight. The obtained stems were pre‐carbonized at 450 °C under Ar gas atmosphere for 3 h to eliminate the presence of organic contaminants. KOH was utilized to activate the carbonized black mass in 1:3 weight ratio. The homogenous mixture was ground well and ramped at 5 °C min^−1^ for 1 h heat‐treatment at 900 °C in Ar atmosphere. 1 M HCl and DI water were utilized to clean the activated powder to eradicate any metal impurities and attain a neutral pH. Thus, activated porous carbon nanosheets were denoted as “activated carbon”. Also, the pre‐carbonized carbon without any activation was labeled “non‐activated carbon” for comparison.

### Electrode Preparation and Device Fabrication

Active material, carbon black (super P C65), and polyvinylidene fluoride (PVDF) binder were mixed in the ratio of 80:10:10 wt. % in *N*‐methyl‐2‐pyrrolidone solvent for the preparation of electrodes. These electrodes were further analyzed for electrochemical performance. The above mixture was ground to form homogenous slurry and coated onto the carbon‐coated aluminum foil followed by drying at 80 °C overnight. Followed by which, 12 mm diameter electrodes (Cathode) were punched, and a Swagelok cell assembly was designed with lithium foil counter electrode and glassy fiber separator in 1 M LiPF_6_ electrolyte EC:DEC solvent.

For Li—C hybrid capacitor fabrication, “Activated carbon” was chosen as the cathode with commercial graphite as the anode in the same electrolyte. Graphite anode electrodes were made by the same procedure mentioned above on the Cu foil current collector. Li metal foil (Li: commercial graphite,1:10) was placed manually over the anode as lithium source. Further, cathode to the anode ratio was maintained at 4:1, with the active mass loading of the cathode to be 4.0 mg cm^−2^ and anode to be 1.0 mg cm^−2^. The cathode half cell was tested between 2.0–4.5 V while the commercial graphite anode half cell was electrochemically tested within 0.01–3.0 V. Further, the full cell was studied between 2.0–4.5 V by employing cyclic voltammetry (CV), galvanostatic charge–discharge (CD), rate capability, cyclic stability, and electrochemical impedance (EIS) techniques.

Additionally, the activated carbon was also explored for its supercapacitor performance. For supercapacitor performance analysis, the electrochemical tests were performed with active material coated Ni mesh, saturated calomel electrode, and platinum mesh as the working, reference, and counter electrodes in a three‐electrode setup with 6 M KOH electrolyte. Further, a symmetric supercapacitor was designed with an active material loading of 5.0 mg cm^−2^ on each electrode in 1 M TEABF_4_/ACN electrolyte. The equations employed for the calculation of specific capacitance, energy density, and power density in both supercapacitor and LIHCs are mentioned in detail in the Supporting Information file.

## Conflict of Interest

The authors declare no conflict of interest.

## Supporting information

Supporting Information

## Data Availability

The data that support the findings of this study are available from the corresponding author upon reasonable request.
