# Supplementary material for: A High‐Energy Density Li‐Ion Hybrid Capacitor Fabricated from Bio‐Waste Derived Carbon Nanosheets Cathode and Graphite Anode
Source: Glob Chall. 2022 Aug 9;6(10):2200082. doi: 10.1002/gch2.202200082 (PMC9581786; doi:10.1002/gch2.202200082)
Supplement: Supplementary file 1 — Supporting Information [file GCH2-6-2200082-s001.pdf]

Global
Challenges
Open Access

Supporting Information

for *Global Challenges,* DOI: 10.1002/gch2.202200082

A High-Energy Density Li-Ion Hybrid Capacitor Fabricated from Bio-Waste Derived Carbon Nanosheets Cathode and Graphite Anode

Katchala Nanaji, Samhita Pappu, Srinivasan Anandan, and Tata N. Rao**

A High-energy density Li-ion hybrid capacitor fabricated from bio-waste derived carbon nanosheets cathode and graphite anode

*Katchala Nanaji *, Samhita Pappu , Srinivasan Anandan , and Tata N Rao **

Centre for Nanomaterials, Centre for Materials Characterization & Testing,

International Advanced Research Centre for Powder Metallurgy and New Materials (ARCI)

Hyderabad-500005, India.

Corresponding Authors: nanaji@arci.res.in ; tata@arci.res.in; Tel: +91-40-24452582

Supplementary information

Material characterization

Crystal structure analysis of the pre-carbonized carbon and activated porous carbon nanosheets were carried out by the Rigaku X-ray Diffractometer (smart lab 9kW) with Cu-Kα radiation (λ = 1.54 Å) in a 2θ range of 10-70°. Surface morphological studies were adapted by Field emission scanning electron microscope FESEM (Zeiss Gemini 500). Microstructural analysis was done by employing JEOL JEM 2100FX high-resolution transmission electron microscopy (HRTEM), and further Raman spectrometer from Horiba with an Ar-ion laser of 514 nm was employed to study the graphitic and disorder properties of the developed carbon materials within a range of 900-3000 cm^{-1}. The specific surface area (SSA) of both the pre-carbonized and activated carbon materials were confirmed by ASAP 2020 BET surface area and pore size analyzer. Additionally, X-ray photoelectron spectroscopy (XPS) by ESCA Omnicron system with Mg Kα as the excitation source was used to identify the various functional moieties attached to the surface of the activated porous carbon.

Specific capacitance in a three-electrode system was calculated from the charge-discharge (CD) profiles using the equation:[1]

$$C_{sp,3E} = \frac{I \times t}{m \times \Delta V} \qquad \text{(eq. S1)}$$

Where, I is the current density in $A\ g^{-1}$, t is the discharge time in *'s'*, m is the active material loading in *'mg'*, and ΔV is the potential window in *'V'*.

For the symmetric supercapacitor, gravimetric specific capacitance was obtained by the equation:

$$C_{sp,2E} = 2 \times \frac{I \times t}{m \times \Delta V} \qquad \text{(eq. S2)}$$

Where, *'m'* is the mass of single electrode.

Further, the energy and power density of the supercapacitor were calculated by using the following equations:

$$E = \frac{1}{2 \times 3.6} C_{cell} \Delta V^2 \qquad \text{(eq. S3)}$$

$$P = \frac{E}{t} \times 3600 \qquad \text{(eq. S4)}$$

Where, $C_{cell} = Csp,_{2E}/4$, E is the energy density in $Wh\ kg^{-1}$ and P is the power density in $W\ kg^{-1}$ respectively.

For LIHC, the specific capacitance of the formulated asymmetric device was obtained based on the following equation:[2]

$$C_{sp} = \frac{I \times t}{m\ (V_{max} - V_{min})} \qquad \text{(eq. S5)}$$

Where, I is the current density in $A\ g^{-1}$, t is the discharge time in *'s'*, m is the active material mass of both cathode and anode put together in *'mg'*, and V_{max} and V_{min} are the potentials at the starting and ending of the discharge process in *'V'*.

Similarly, energy and power densities were accessed based on the following equations:

$$E = \frac{1}{2}\, C\, (V_{max} + V_{min})\, (V_{max} - V_{min}) \qquad \text{(eq. S6)}$$

$$P = \frac{E}{t} \qquad \text{(eq. S7)}$$

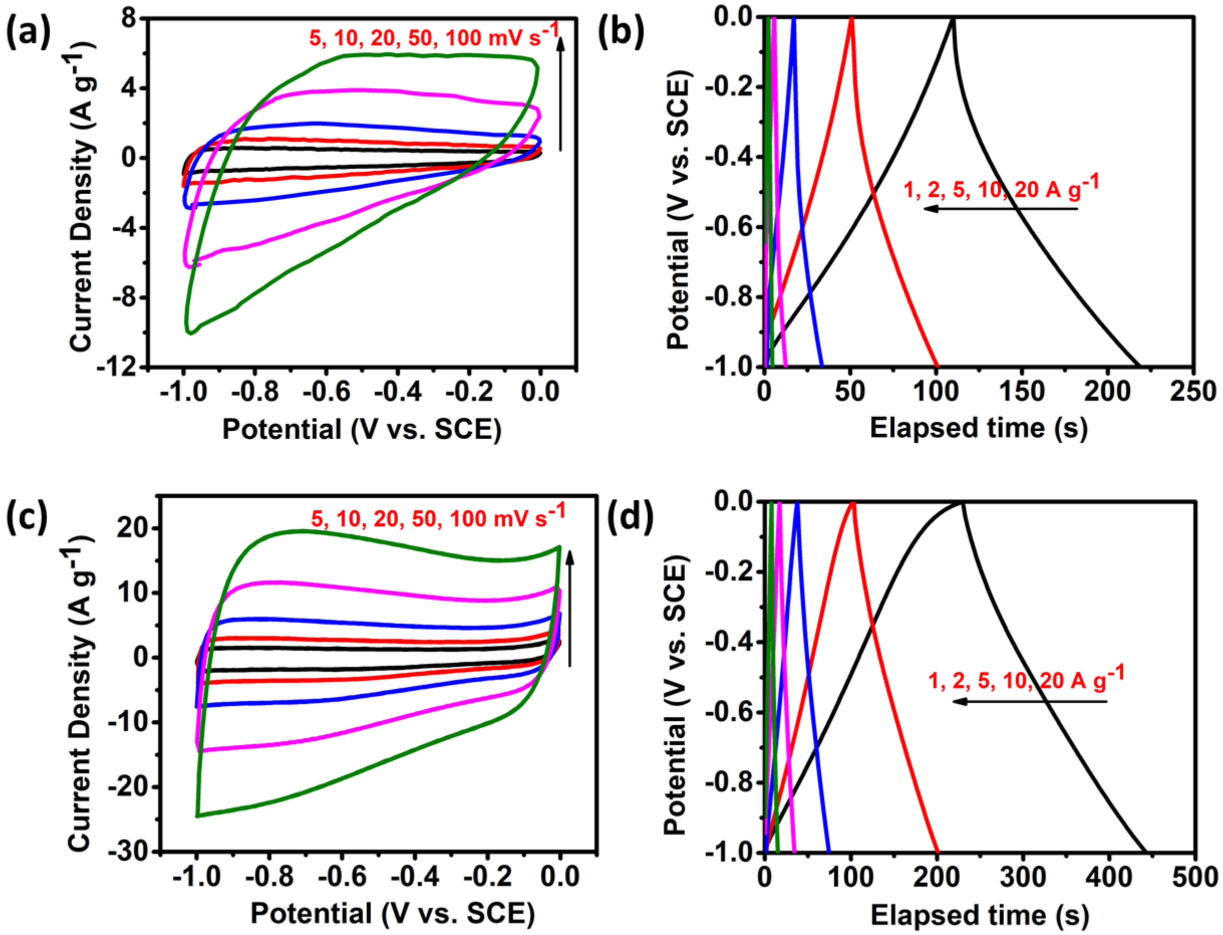

Figure S1. Cyclic voltammetry at different scan rates and charge-discharge analysis at different current densities of (a,b) non-activated (Mendeley), and (c,d) activated carbon

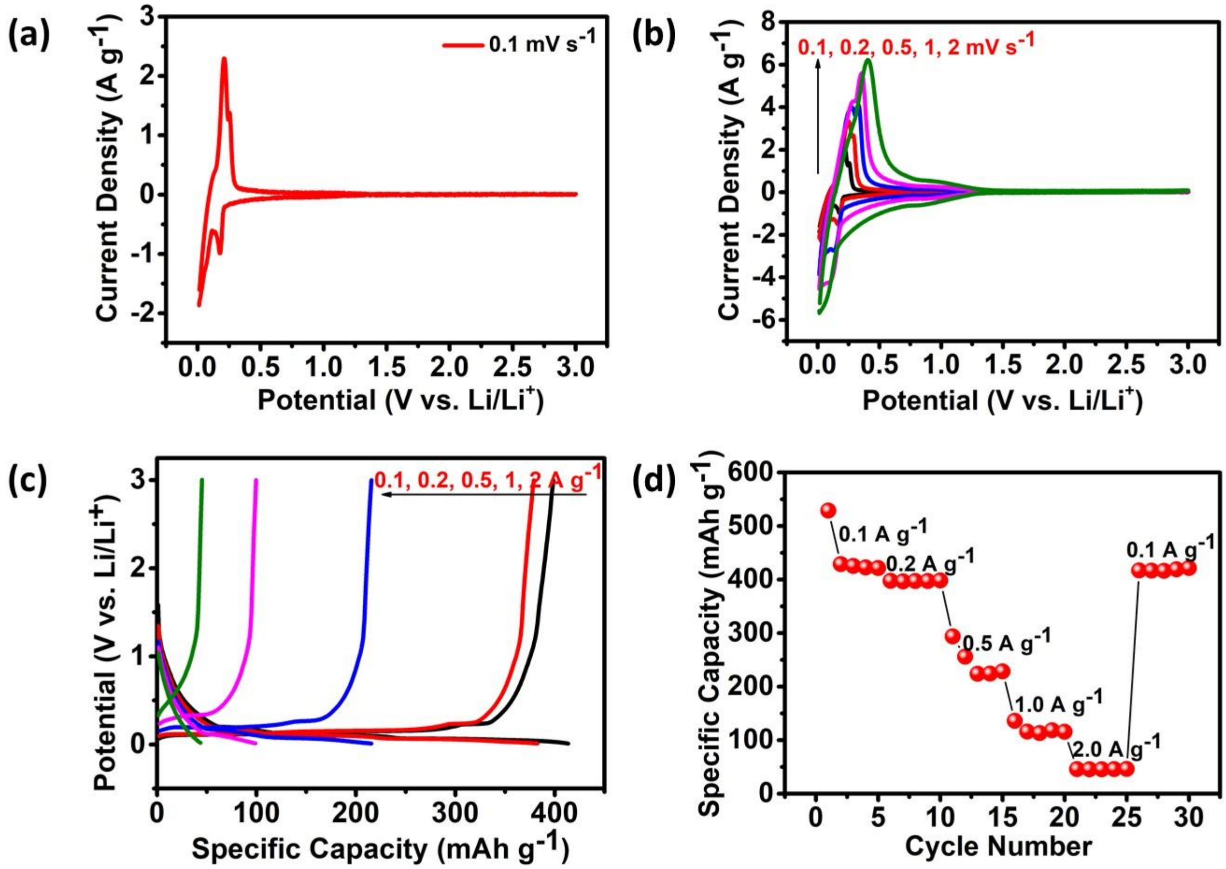

Figure S2. CV at 0.1 mV s^{-1} scan rate, (b) CV at different increasing scan rates, (c) CD at different current densities, and (d) rate capability analysis of commercial graphite anode vs. Li/Li^+ ions

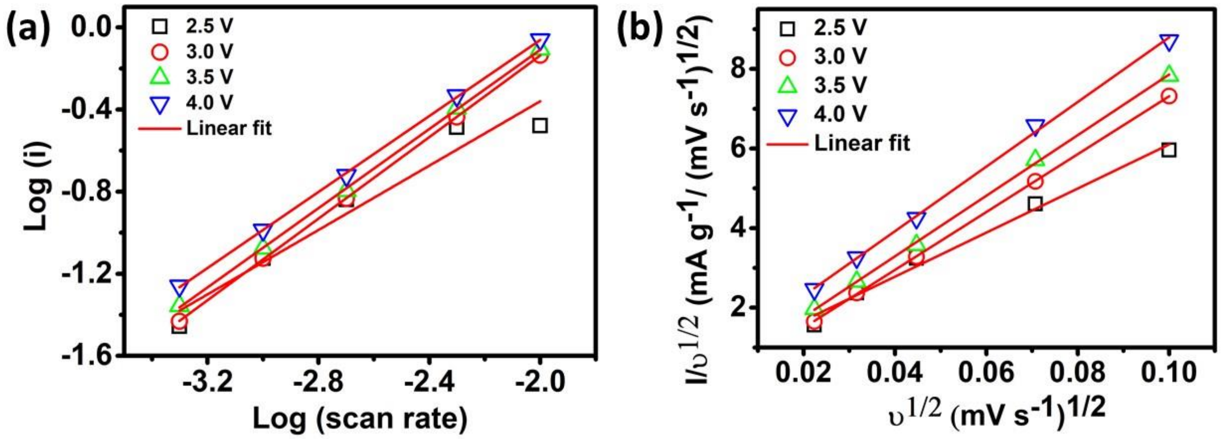

Figure S3. (a) Plot of log (i) vs. log (scan rate), and (b) $I/v^{1/2}$ vs. $v^{1/2}$ at different voltages of 2.5, 3.0, 3.5, 4.0 V respectively.

References:

1 K. Nanaji, B. V. Sarada, U. V. Varadaraju, T. N Rao and S. Anandan, *Renew. Energy*, 2021, **172**, 502–513.

2 C. Li, X. Zhang, K. Wang, X. Sun and Y. Ma, *J. Power Sources*, 2018, **400**, 468–477.
